# Transplantation of human bone marrow mesenchymal stromal cells reduces liver fibrosis more effectively than Wharton’s jelly mesenchymal stromal cells

**DOI:** 10.1186/s13287-017-0595-1

**Published:** 2017-06-13

**Authors:** Mathiyazhagan Rengasamy, Gurbind Singh, Noor Atiqah Fakharuzi, Siddikuzzaman, Sudha Balasubramanian, Priyanka Swamynathan, Charan Thej, Gopinath Sasidharan, Pawan Kumar Gupta, Anjan Kumar Das, Ahmad Zuhairi Abd Rahman, Kamal Shaik Fakiruddin, Lim Moon Nian, Zubaidah Zakaria, Anish S. Majumdar

**Affiliations:** 1 Stempeutics Research Pvt Ltd, Akshay Tech Park, EPIP Zone, Phase-1, Whitefield, Bangalore, 560066 Karnataka India; 2 Hematology Unit, Cancer Research Centre, Institute for Medical Research, Jalan Pahang, 50588 Kuala Lumpur, Malaysia; 3 Department of Life Sciences, International Medical University, Bukit Jalil, 57000 Kuala Lumpur, Malaysia; 4 Manipal University, Manipal, Karnataka India; 5 Department of Surgery, Taylor’s University School of Medicine, Selangor, Subang Jaya Malaysia

**Keywords:** Human BM- and WJ-MSCs, Liver fibrosis, MMPs, Angiogenesis

## Abstract

**Background:**

Mesenchymal stromal cells (MSCs) from various tissues have shown moderate therapeutic efficacy in reversing liver fibrosis in preclinical models. Here, we compared the relative therapeutic potential of pooled, adult human bone marrow (BM)- and neonatal Wharton’s jelly (WJ)-derived MSCs to treat CCl_4_-induced liver fibrosis in rats.

**Methods:**

Sprague-Dawley rats were injected with CCl_4_ for 8 weeks to induce irreversible liver fibrosis. Ex-vivo expanded, pooled human MSCs obtained from BM and WJ were intravenously administered into rats with liver fibrosis at a dose of 10 × 10^6^ cells/animal. Sham control and vehicle-treated animals served as negative and disease controls, respectively. The animals were sacrificed at 30 and 70 days after cell transplantation and hepatic-hydroxyproline content, histopathological, and immunohistochemical analyses were performed.

**Results:**

BM-MSCs treatment showed a marked reduction in liver fibrosis as determined by Masson’s trichrome and Sirius red staining as compared to those treated with the vehicle. Furthermore, hepatic-hydroxyproline content and percentage collagen proportionate area were found to be significantly lower in the BM-MSCs-treated group. In contrast, WJ-MSCs treatment showed less reduction of fibrosis at both time points. Immunohistochemical analysis of BM-MSCs-treated liver samples showed a reduction in α-SMA^+^ myofibroblasts and increased number of EpCAM^+^ hepatic progenitor cells, along with Ki-67^+^ and human matrix metalloprotease-1^+^ (MMP-1^+^) cells as compared to WJ-MSCs-treated rat livers.

**Conclusions:**

Our findings suggest that BM-MSCs are more effective than WJ-MSCs in treating liver fibrosis in a CCl_4_-induced model in rats. The superior therapeutic activity of BM-MSCs may be attributed to their expression of certain MMPs and angiogenic factors.

## Background

Liver fibrosis/cirrhosis is a major health problem worldwide and, among the 1.4 million liver disease deaths occurring each year, 55% of these are attributed to liver cirrhosis [1, 2]. Liver fibrosis/cirrhosis-related mortality has been steadily increasing worldwide, as has alcohol consumption, and the prevalence of hepatitis B, C, and diabetes [3]. It has been reported that almost one-fifth (18.3%) of global liver fibrosis/cirrhosis deaths occur in India [3]. Currently, there is no effective treatment available to cure liver fibrosis/cirrhosis. Liver transplantation remains the only option but this is hindered by a lack of donor organs and immune-rejection. Transplantation of adult hepatocytes is another alternative and has been used in clinical studies, predominantly in hereditary metabolic disorders [4, 5] or as a bridging therapy for patients awaiting liver transplantation [6]. Again, a major limitation for broader use of hepatocyte transplantation is the lack of availability of sufficient numbers of primary human hepatocytes.

Understandably, there is a critical need to find an effective alternative therapy for this serious life-threatening disease. Stem cell-based therapy has been considered a potential alternative to liver transplantation. The encouraging advances in stem cell research have provided hope that these cells could be used for the treatment of the end-stage chronic liver diseases. Among various types of stem cells, mesenchymal stromal cells (MSCs) are the preferred cell type due to their easy isolation, high expandability, multilineage differentiation potential, and paracrine activity [7]. Not only can they differentiate into mesodermal lineage but they also have the ability to differentiate into hepatocyte-like cells in vitro and in vivo [8, 9]. MSCs secrete a broad spectrum of growth factors such as hepatocyte growth factor (HGF), vascular endothelial growth factor (VEGF), nerve growth factor (NGF), angiopoetin-1 (Ang-1), insulin-like growth factor (IGF-1) and fibroblast growth factor (FGF-2). Among them HGF, NGF, and VEGF have been identified as important growth factors involved in the regenerative process via antifibrotic, antiapoptotic, progenitor cell proliferation, and neoangiogenesis effects [10]. MSCs have immunosuppressive and anti-inflammatory properties such as inhibitory effects on dendritic cells, natural killer (NK) cells, Th1 cell proliferation, and activation of M2 macrophages and Th2 cells. These effects are mediated via production of prostaglandin E2 (PGE2), indoleamine 2,3-dioxygenase (IDO), nitric oxide (NO), and secretion of anti-inflammatory interleukins such as IL-10 [11–13]. These characteristics make MSCs a potential candidate for treating end-stage liver diseases.

Several human clinical trials have been performed using various types of MSCs through different routes of delivery to see the improvement in clinical end-points of liver function in patient with liver fibrosis/cirrhosis and liver failure [14]. Infusion of autologous bone marrow (BM)-derived MSCs (BM-MSCs) through intrahepatic artery delivery [15] showed improvements in liver biochemical parameters and in histological evaluation of fibrosis. Recently, the beneficial effect of allogeneic umbilical cord (UC)-derived MSCs and BM-MSCs through peripheral vein infusion in patients with liver cirrhosis has been shown [16]. The authors demonstrated improvement in liver function parameters such as total bilirubin, serum albumin, and liver enzymes, as well as model for end-stage liver disease (MELD) score, though all the parameters were not found to be significant. These studies indicate that stem cells including allogeneic MSCs derived from different tissues may provide clinical benefit to these patients, although larger clinical trials with higher numbers of subjects is warranted.

MSCs derived from various tissue sources such as bone marrow, Wharton’s jelly (WJ), adipose tissue, umbilical cord blood, and placenta have been tested in various preclinical models of fibrosis [17]. Among these, BM-MSCs and WJ-MSCs have been thoroughly characterized for their phenotypic expression, cytokine secretion, and immunomodulatory properties. In addition, their antifibrotic activity has been shown in various in vitro and in vivo models [18, 19]. However, the most efficacious tissue source of MSCs for the treatment of liver fibrosis/cirrhosis has not been identified. In this manuscript, we investigated various properties of pooled populations of adult human BM-MSCs and neonatal human WJ-MSCs that may play a critical role in treating liver fibrosis. We observed certain key differences between the two cell types with regards to their angiogenic factor(s) secretion, matrix metalloprotease (MMP) expression, and ability for collagen degradation in vitro. These differences led us to determine the relative therapeutic potential of BM-MSCs and WJ-MSCs in a CCl_4_-induced preclinical model of liver fibrosis in rats.

## Methods

### Isolation and expansion of BM-MSCs and WJ-MSCs

Bone marrow mononuclear cells (BMMNCs) from three independent donor aspirations, obtained after appropriate informed consent, were separated by the Ficoll density gradient method (1.077 g/ml density). BMMNCs accumulated at the Ficoll-plasma interphase were isolated by carefully aspirating the buffy coat. The buffy layer was then transferred into a fresh 50-ml centrifuge tube, to which an equal volume of culture medium was added, and centrifuged at 1200 rpm for 10 min. The resulting pellet was resuspended with culture medium comprising of 10 ml KO-DMEM (Gibco), 10% fetal bovine serum (FBS; Hyclone), 100 U/ml penicillin (Gibco), 100 μg/ml streptomycin, and 2 mM GlutaMAX™ (Gibco) and gently mixed to obtain a single cell suspension. To 18 ml of freshly prepared complete culture medium in a T75 flask, 2 ml of MNC suspension was added, transferred to a 5% CO_2_ incubator, and left undisturbed for 48 h. After 48 h, the culture flasks were screened for adherent cells and the nonadherent cells were removed carefully. The cultures were supplemented with 15 ml of fresh medium per flask and the flasks were returned to the CO_2_ incubator. The medium was replenished every 48 to 72 h. Cells were cultured until they achieved 70-80% confluency. Subsequently, BM-MSCs from the three donors were pooled in equal proportions and expanded in culture. The cells were seeded at a density of 1000 cells/cm^2^, supplemented with 2 ng/ml recombinant human basic fibroblast growth factor (bFGF; Sigma-Aldrich) from passage 2 (P2) onwards, and expanded in 10 CellSTACK up to P5 as described previously [20, 21]. The cells from P5 were used for further in vitro and in vivo experiments in this study.

For WJ-MSC isolation and expansion, fresh umbilical cords were collected from full-term births after obtaining written informed consent following approval of the ethics committee. After a brief rinse with normal saline (0.9% w/v, sodium chloride), cords were given a quick rinse in 70% isopropanol followed by three washes using sterile DPBS (Gibco). The umbilical cord vein and arteries were removed, and the exposed mesenchymal tissue was cut into small pieces of 1–2 mm before placing them in the tissue culture dish [22]. The explants were cultured in KO-DMEM (Gibco), 10% FBS (Hyclone), 100 U/ml Penicillin (Gibco), 100 μg/ml streptomycin, and 2 mM GlutaMAX™ (Gibco). The MSCs obtained from individual cords were expanded up to P2 using an initial seeding density of 3000 cells/cm^2^ as standardized and reported in our earlier publication [22]. Cells obtained from three individual cords were pooled at P3, when 2 ng/ml bFGF (Sigma-Aldrich) was introduced into the culture medium. The cells were further expanded in culture medium with 2 ng/ml bFGF up to P6. The WJ-MSCs harvested at P6 were used for further in vitro and in vivo experiments in this study.

### Characterization of BM-MSCs and WJ-MSCs

Both BM-MSCs and WJ-MSCs were harvested at 80–90% confluence and resuspended in DPBS at a cell density of 1 × 10^6^ cells/ml. The following marker expressions were analyzed for MSC identification: CD73 (PE), CD90 (PE), and CD105 (PE). To rule out the presence of hematopoietic cells, we used CD14 (FITC), CD19 (FITC), CD34 (FITC), CD45 (FITC), and HLA-DR (FITC) (BD Pharmingen, San Diego, USA) antibodies. FITC- or PE-conjugated mouse IgGs (BD Pharmingen, San Diego, USA) were used as isotype controls. The fluorescence intensity of MSCs was analyzed by flow cytometry (Guava easyCyte™ flow cytometers, Millipore, CA, USA). The adipogenic, osteogenic, and chondrogenic differentiation ability of BM-MSCs and WJ-MSCs was also evaluated according to a procedure described earlier [21–23].

### Comparative molecular analysis of pooled BM-MSCs and WJ-MSCs

Total cellular RNA was isolated using an RNeasy mini kit (Qiagen). The RNA samples were treated with DNase I (Ambion) and reverse-transcribed into cDNA using a high-capacity cDNA reverse transcription kit (Applied Biosystems) according to the manufacturer’s instructions. The cDNAs were amplified by using gene specific primers as shown in Table 1. A reverse transcriptase negative blank of each sample and a no-template blank served as negative controls. Gene expression was normalized to the housekeeping gene β-actin. Human VEGF, human HGF, and transforming growth factor (TGF)-β in the conditioned medium (CM) were estimated using enzyme-linked immunosorbent assay (ELISA) kits (R&D Systems) according to the manufacturer’s instructions.

**Table 1:** Primer sequences to amplify cDNA using real time polymerase chain reaction

Genes	Primers	Primer squences	Primer Size (bp)	Tm	Product size (bp)
MMP-1	Forward Primer	5′-AAGGCCAGTATGCACAGCTT-3′	20	58.3	480
	Reverse Primer	5′-TGCTTGACCCTCAGAGACCT-3′	20		
MMP-2	Forward Primer	5′-TTTCCATTCCGCTTCCAGGGCAC-3′	23	63	253
	Reverse Primer	5′-TCGCACACCACATCTTTCCGTCACT-3′	25		
MMP-3	Forward Primer	5′-GGCTTTCCCAAGCAAATAGC-3′	20	57.3	205
	Reverse Primer	5′-GTGCCCATATTGTGCCTTCT-3′	20		
MMP-7	Forward Primer	5′-TCCAACCTATGGAAATGGAGA-3′	21	57.3	196
	Reverse Primer	5′-GGAGTGGAGGAACAGTGCTT-3′	20		
MMP-8	Forward Primer	5′-TCTGCAAGGTTATCCCAAGG-3′	20	57.3	154
	Reverse Primer	5′-ACCTGGCTCCATGAATTGTC-3′	20		
MMP-9	Forward Primer	5′-CCTGCCAGTTTCCATTCATC-3′	20	57.3	455
	Reverse Primer	5′-GCCATTCACGTCGTCCTTAT-3′	20		
MMP-12	Forward Primer	5′-ACAGATGATGGACCCTGGTT-3′	20	57.3	392
	Reverse Primer	5′-AGAGTCAAGCAAGAATGGACAA-3′	22		
MMP-13	Forward Primer	5′-AACATCCAAAAACGCCAGAC-3′	20	55.3	166
	Reverse Primer	5′-GGAAGTTCTGGCCAAAATGA-3′	20		
MMP-15	Forward Primer	5′-AGGAGACACAGCGTGGAGAC-3′	20	60	514
	Reverse Primer	5′-TTGCAGTAAAGCAGGACACG-3′	20		
MMP-16	Forward Primer	5′-GACATGCTCTGGGATTGGAG-3′	20	57.3	217
	Reverse Primer	5′-TCATTTTTCCTTGGGTCAGC-3′	20		
MMP-24	Forward Primer	5′-GAACCTGTGGGCAAGACCTA-3′	20	58.3	213
	Reverse Primer	5′-TGACAACCAGAAACTGAGCG-3′	20		

### Inhibition of hepatic stellate cells activation

Hepatic stellate cells (HSCs; LX-2 line, Cat. No. SCC064, Millipore) were seeded at a density of 3000 cells/cm^2^. At 50% confluence, the cells were activated with 2% FBS (in KO-DMEM) medium containing 10 ng/ml human TGFβ-1 (Cat. No. 100-21, Peprotech) for 48 h. Next, the medium was replaced with conditioned medium (CM) of BM-MSCs or WJ-MSCs, and diluted to 50% in serum-free medium. The HSCs were incubated for 72 h and processed further for collagen estimation using the Sircol™ Soluble Collagen Assay kit (Cat. No. S1000, Biocolor), as per the manufacturer’s instructions. The HSCs were also plated in chamber slides and tested for the expression of α-smooth muscle actin (SMA) by immunofluorescence assay. Fluorescent images were captured using a Nikon-Eclipse-90i microscope (Nikon) and Image-Pro AMS version 6.0 software.

### Animals

In this study, male Sprague-Dawley rats were obtained from Harlan laboratories (Indianapolis, Indiana, USA). Rats were maintained in a controlled environment at 22 ± 3 °C temperature, 50 ± 20% humidity, and a light/dark cycle of 12 h each. The experimental protocol was approved by the Institutional Animal Ethics Committee (IAEC) of Syngene International animal facility (Syngene/IAEC/511/06-2014) and the experiments were conducted in the same facility.

### Establishment of the CCl_4_-induced liver fibrosis model

**Fig. 1:**
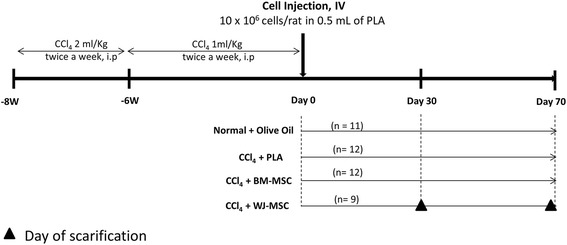
Study design: Rats were treated with CCl_4_ (1:1 ratio, CCl_4_:olive oil) or olive oil alone for 8 weeks to induce liver fibrosis/cirrhosis. After 8 weeks of CCl_4_ injection, rats were randomly assigned into three groups and injected with vehicle PlamaLyte﻿-A(PLA) alone, BM-MSCs (10 × 10^6^ cells/rat) or WJ-MSCs (10 × 10^6^ cells/rat). Rats were sacrificed at days 30 and 70 after MSCs transplantation. *BM-MSCs﻿;* bone marrow-derived mesenchymal stromal cells, *IV*; Intravenous, *i.p*; intraperitoneal, *PLA*; Plasma-Lyte A, *WJ-MSCs﻿*; Wharton’s jelly-derived mesenchymal stromal cells

Among the various types of experimental liver fibrosis/cirrhosis models, CCl_4_-induced method appears to be the most widely applied [24, 25]. In this experiment, liver fibrosis/early cirrhosis was induced in Sprague-Dawley rats by intraperitoneal injection of CCl_4_ as published by Issa et al. [24] with slight modifications after internal validation. CCl_4_ was administered at a dose of 2 ml/kg body weight (CCl_4_:olive oil = 1:1) twice weekly for an initial 2 weeks, followed by 1 ml/kg body weight twice weekly for the next 6 weeks (Fig. 1). After 48 h from the last CCl_4_ injection, blood was collected, and the serum was separated and analyzed for aspartate transaminase, alanine transaminase, and total bilirubin by an automated analyzer (EM-360, Erba Mannheim, Germany) according to the manufacturer’s instructions. Based on the results of the alanine transaminase, aspartate transaminase, and total bilirubin analysis, animals were randomly assigned into three groups: vehicle control (*n* = 12) and BM-MSCs (*n* = 12) and WJ-MSCs (*n* = 9) treatment groups. Rats that had not been treated with CCl_4_ (*n* = 11) served as normal controls.

### MSCs administration

Cryopreserved pooled human BM-MSCs or WJ-MSCs were thawed at 37 °C in a water bath, centrifuged, and the pellet was resuspended in PlasmaLyte A (vehicle) at a dose of 10 × 10^6^ BM-MSCs or WJ-MSCs in 0.5 ml vehicle solution. The cells were administered through the tail vein at an infusion rate of 150 μl/min. A separate group of rats received only vehicle (0.5 ml) which served as a control. Rats were sacrificed at days 30 and 70 after cell treatment using CO_2_ asphyxiation. On day 30, rats from the vehicle (*n* = 5), BM-MSCs (*n* = 5) and WJ-MSCs (*n* = 4) treatment groups were sacrificed. The remaining rats were sacrificed at day 70. During necropsy, samples of liver tissues were collected for liver hydroxyproline content analysis, and histopathological and immunohistochemistry evaluation.

### CM-DiI labeling of BM-MSCs and WJ-MSCs

The BM-MSCs or WJ-MSCs were labeled using CM-DiI fluorescent dye (Invitrogen) in order to track them in vivo in rats. Briefly, the cells were resuspended in KO-DMEM (Gibco) along with 5 μM CM-DiI and incubated for 30 min at 37 °C. The excess dye was removed by washing with DPBS, and the cells were resuspended in Plasmalyte A and injected at a dose of 10 × 10^6^ cells/rat through the tail vein. Animals received vehicle alone to serve as normal controls. The DiI-labeled cells were confirmed ex vivo in liver tissues by fluorescence microscopy after 30 days of cell administration.

### Quantitative analysis of hepatic hydroxyproline content

The liver hydroxyproline content was quantitated using a hydroxyproline assay kit (QuickZyme Biosciences, Leiden, Netherlands) according to the manufacturer’s instructions. Briefly, the liver tissue (100 mg) was homogenized in 1.5 mL distilled water containing protease inhibitors. Tissue samples were then hydrolyzed at 110 °C for 16 h for oxidation of free hydroxyproline. The hydroxyproline content was assessed by spectrophotometry (Thermo Scientific) at 570 nm.

### Histopathological analysis

Liver specimens collected in 10% neutral buffered formalin (NBF) were processed, embedded in paraffin, sectioned (5 μm thick), and stained with hematoxylin and eosin (H&E), Masson’s trichrome (MTC), and Picrosirius red (PSR) for histological examination. The degree of hepatic fibrosis was assessed according to the Ishak modified scoring system [26].

Morphometric analysis was performed using digitally captured serial images (*n* = 5) of Sirius red stained sections using Leica software, and the collagen stained area was calculated in a blinded manner.

### Immunohistochemical analysis of liver tissues

Immunohistochemical studies were performed on paraffin-embedded liver tissue sections (5 μm thickness) using rat α-SMA antibody (1:1000; Sigma-Aldrich, cat. no. A5228), rat Ki-67 antibody (5 μg/ml; Abcam, cat. no. ab15580), rat EpCAM antibody (1:160; Abcam, cat. no. ab71916), human MMP1 antibody (1:100; Abcam, cat. no. ab52631), and HRP-conjugated secondary antibodies (GBI labs, cat no. D39-18). The immunoreactive product was visualized by adding substrate-chromogen diaminobenzidine (DAB) and counterstained by Mayer’s hematoxylin solution. Stained slides were analyzed by light microscopy (Olympus BX51, Tokyo, Japan).

### Immunofluorescence analysis of liver tissues

For immunofluorescence analysis, 5-μm thicknesses of OCT frozen tissue sections were fixed in 4% paraformaldehyde and stained with mouse anti-human CD105 antibody (1:25; BD Pharmingen, cat. no. 555690) followed by a secondary goat anti-mouse FITC antibody (Abcam, cat. no. ab6785). All sections were washed three times and costained with a 1:2000 solution of DAPI (Invitrogen). The DiI/CD105^+^ cells were visualized and enumerated in five random areas under a fluorescence microscope.

### Statistical analysis

The data were expressed as mean ± SEM. For the comparison of different treatment groups, the data was analyzed by one-way analysis of variance (ANOVA) followed by Dunnett’s multiple comparison test. The histopathological parameters were analyzed using the Kruskal-Wallis test. The results were considered statistically significant at *p* < 0.05.

## Results

### Characterization of pooled BM-MSCs and WJ-MSCs

**Fig. 2:**
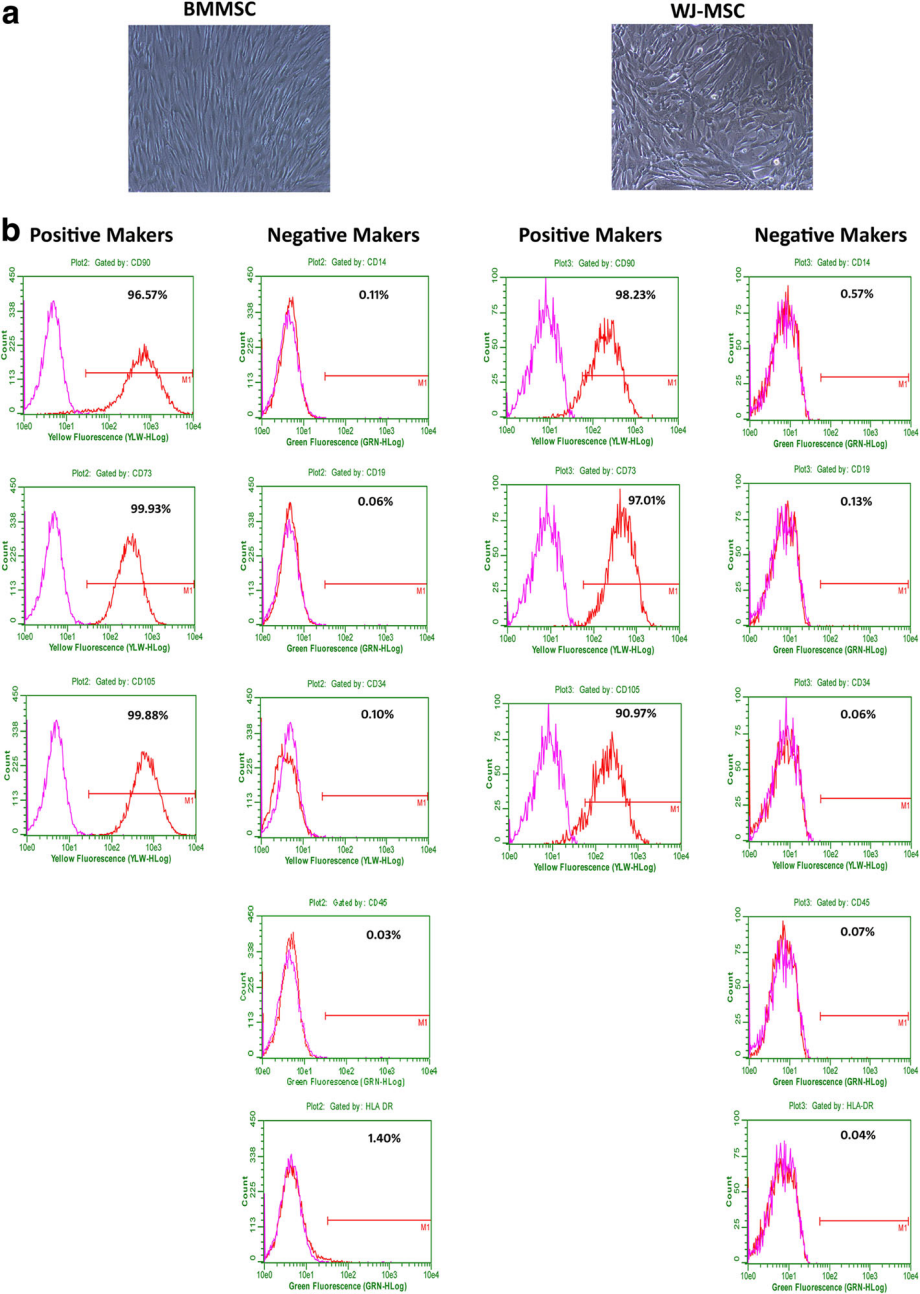
Morphological and immunophenotypic analysis of BM-MSCs and WJ-MSCs. **a** Photomicrographs of BM-MSCs (*left*) and WJ-MSCs (*right*) showing spindle-shaped morphology. **b** Flow cytometric analysis of MSCs positive markers (CD90, CD73, and CD105) and negative markers (CD14, CD19, CD34, CD45, and HLA-DR). *BM-MSCs;﻿* bone marrow-derived mesenchymal stromal cells, *WJ-MSCs;﻿* Wharton’s jelly-derived mesenchymal stromal cells

Both BM-MSCs and WJ-MSCs showed the plastic adherence properties and spindle-shaped morphology which are the primary characteristics of MSCs (Fig. 2a). Flow cytometric analysis revealed that these cells expressed CD73, CD90, and CD105 markers and were negative for the hematopoietic markers CD14, CD19, CD34, and CD45. Both BM-MSCs and WJ-MSCs lacked the expression of HLA-DR (Fig. 2b). The adipogenic, osteogenic, and chondrogenic differentiation of BM-MSCs and WJ-MSCs have been published earlier by us [21, 23].

### Comparative molecular analysis of BM-MSCs and WJ-MSCs

**Fig. 3:**
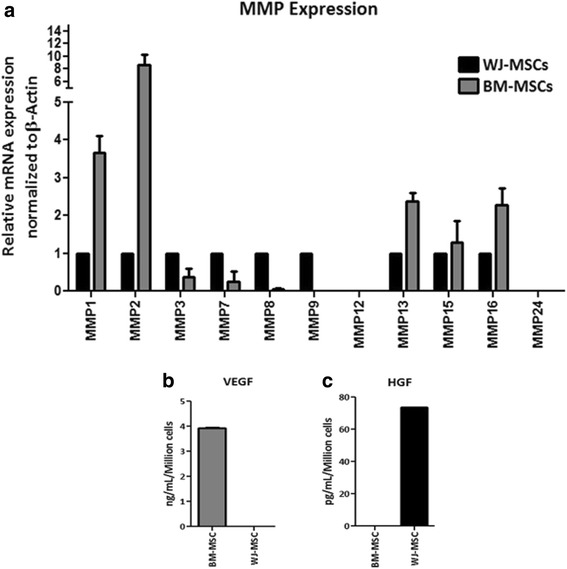
Quantitative reverse transcription polymerase chain reaction analysis of collagen-degrading MMPs and growth factor secretion by BM-MSCs and WJ-MSCs. **a** MMP gene expression analysis showing quantification of various MMPs. **b**, **c** Quantitative analysis of VEGF and HGF secretion from BM-MSCs and WJ-MSCs. *BM-MSCs*; bone marrow-derived mesenchymal stromal cells, *HGF*; hepatocyte growth factor, *MMP*; matrix metalloprotease, *VEGF*; vascular endothelial growth factor, *WJ-MSCs﻿*; Wharton’s jelly-derived mesenchymal stromal cells

The gene expression of MMP1, MMP2, MMP13, MMP15, and MMP16 were observed to be higher in BM-MSCs as compared to the WJ-MSCs. Both cell types exhibited expression of MMP3, MMP7, and MMP8. A higher expression of MMP9 was observed in the WJ-MSCs compared to BM-MSCs, whereas none of the cell types expressed MMP12 or MMP24 genes (Fig. 3a). The secretion of VEGF was only observed with BM-MSCs culture (3.9 ± 0.03 ng/ml/million cells), whereas the WJ-MSCs secreted higher amounts of HGF (73.8 ± 0.03 pg/ml/million cells) (Fig. 3b and c). In addition, we observed that TGF-β1 expression was significantly higher in WJ-MSCs than BM-MSCs (data not shown).

### Effect of BM-MSCs and WJ-MSCs conditioned media on activated HSCs

**Fig. 4:**
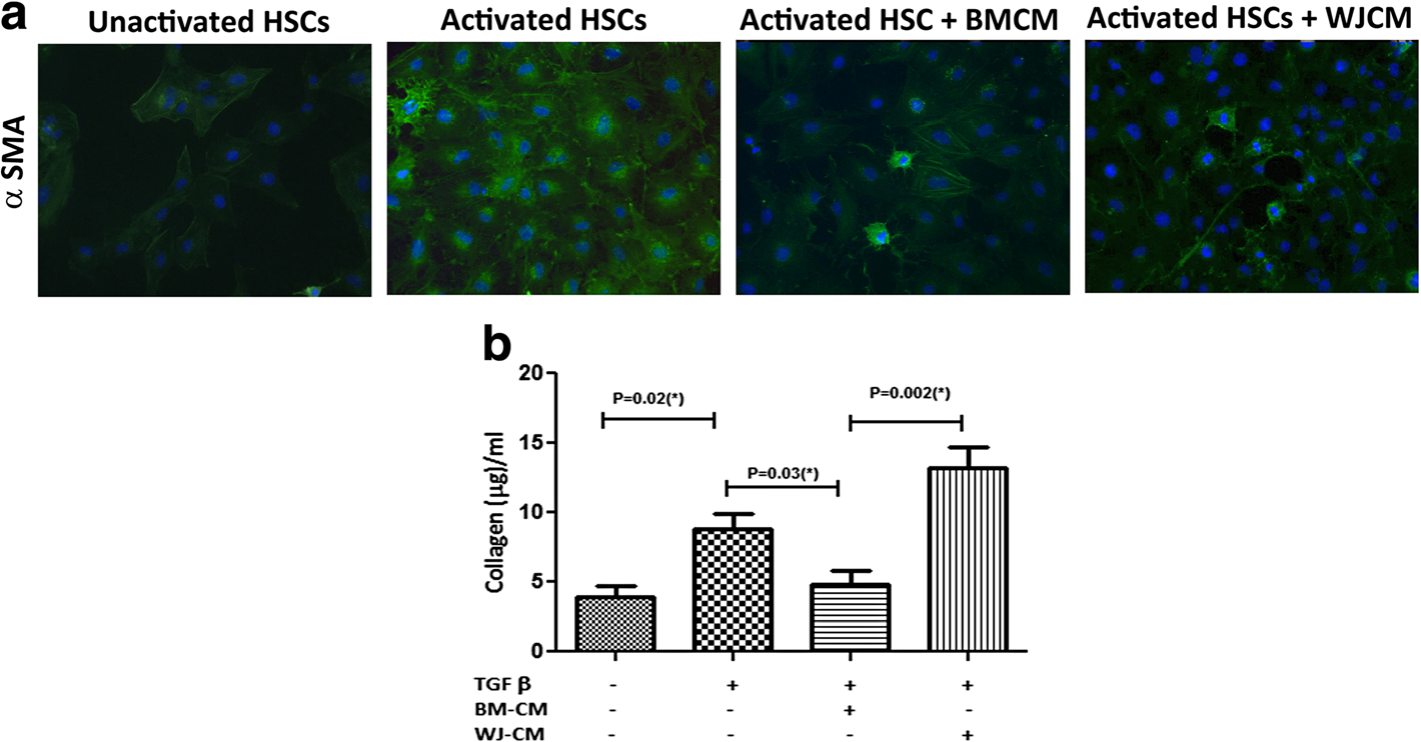
α-Smooth muscle actin expression (*α-SMA*) in the hepatic stellate cells (*HSCs*) under various culture conditions. **a** α-SMA in unactivated HSCs (*left*), HSCs activated with TGF-β1 (*left center*), TGF-β1-activated HSCs treated with BM-MSCs conditioned medium (*BMCM*) (*right center*), and TGF-β1-activated HSCs treated with WJ-MSCs conditioned medium (*WJCM*) (*right*). **b** Quantification of collagen levels on the unactivated HSCs, TGF-β1-activated HSCs, and activated HSCs treated with conditioned medium derived from bone marrow and Wharton’s jelly. Data are shown as mean ± SEM

The expression of α-SMA in HSCs was analyzed by immunocytochemistry which is a quantitative measure of hepatic fibrosis and could be related directly to liver fibrogenesis and indirectly to human liver fibrosis in chronic liver disease. BM-MSCs-CM treatment showed a marked reduction in the α-SMA-positive cells as compared to WJ-MSCs-CM-treated HSCs (Fig. 4a). To determine the effect of both BM-MSCs and WJ-MSCs on collagen synthesis by activated HSCs, conditioned media obtained from both cell types were tested on activated HSCs. As shown in Fig. 4b, BM-MSCs-CM treatment resulted in a significant reduction in collagen synthesis by the activated HSCs compared to the WJ-MSCs-CM-treated cells. These data suggest that BM-MSCs secrete factors necessary to inhibit HSCs activation and collagen deposition in vitro.

### Differential intrahepatic engraftment of DiI-labeled BM-MSCs and WJ-MSCs in the CCl_4_ animal model

A total of sixteen intraperitoneal injections of CCl_4_ were given to Sprague-Dawley rats to develop the liver fibrosis model. For the first 2 weeks, an intraperitoneal injection of CCl_4_ at a dose of 2 ml/kg body weight was given twice weekly followed by 1 ml/kg body weight for an additional 6 weeks (twice weekly) to establish extensive liver fibrosis in Sprague-Dawley rats.

**Fig. 5:**
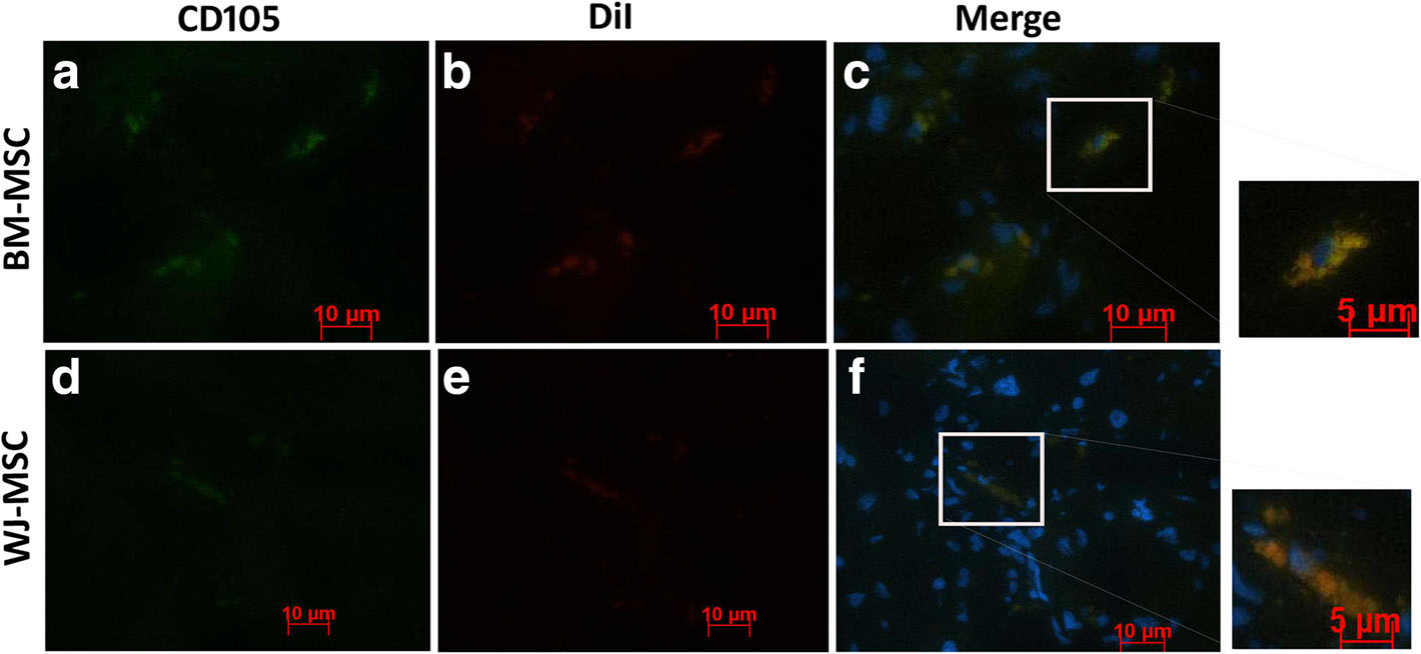
Immunofluorescence analysis for **a**–**c** BM-MSCs and **d**–**f** WJ-MSCs engraftment in the liver tissues of CCl_4_-treated rats after 30 days of cell injection. Representative fluorescence images show colocalization of human CD105 expression (**a**, **d**) and DiI-positive cells (**b**, **e**) in liver tissue sections. The photomicrographs were captured using 40× and 60× (insets) objectives. *BM-MSCs﻿;* bone marrow-derived mesenchymal stromal cells, *WJ-MSCs﻿;* Wharton’s jelly-derived mesenchymal stromal cells

To determine the intrahepatic distribution of BM-MSCs or WJ-MSCs, animals injected with CM-DiI-labeled cells were sacrificed 30 days after cell injection. The number of DiI/CD105^+^ cells were identified in the liver section and counted in five random fields from each slide under 40× magnification. The results indicated that the number of DiI/CD105^+^ cells were higher in BM-MSCs-treated animals (1.8 ± 0.84) than WJ-MSC-treated animals (0.8 ± 1.09). The purpose of costaining the MSCs with human CD105 along with DiI was to rule out the possibility of capturing false signals resulting from dead human cells phagocytosed by rat macrophages. These findings suggest that a greater number of intravenously injected BM-MSCs survived and sporadically colonized in the liver in comparison to WJ-MSCs (Fig. 5).

### Effect of intravenously injected BM-MSCs and WJ-MSCs on hepatic hydroxyproline content

**Fig. 6:**
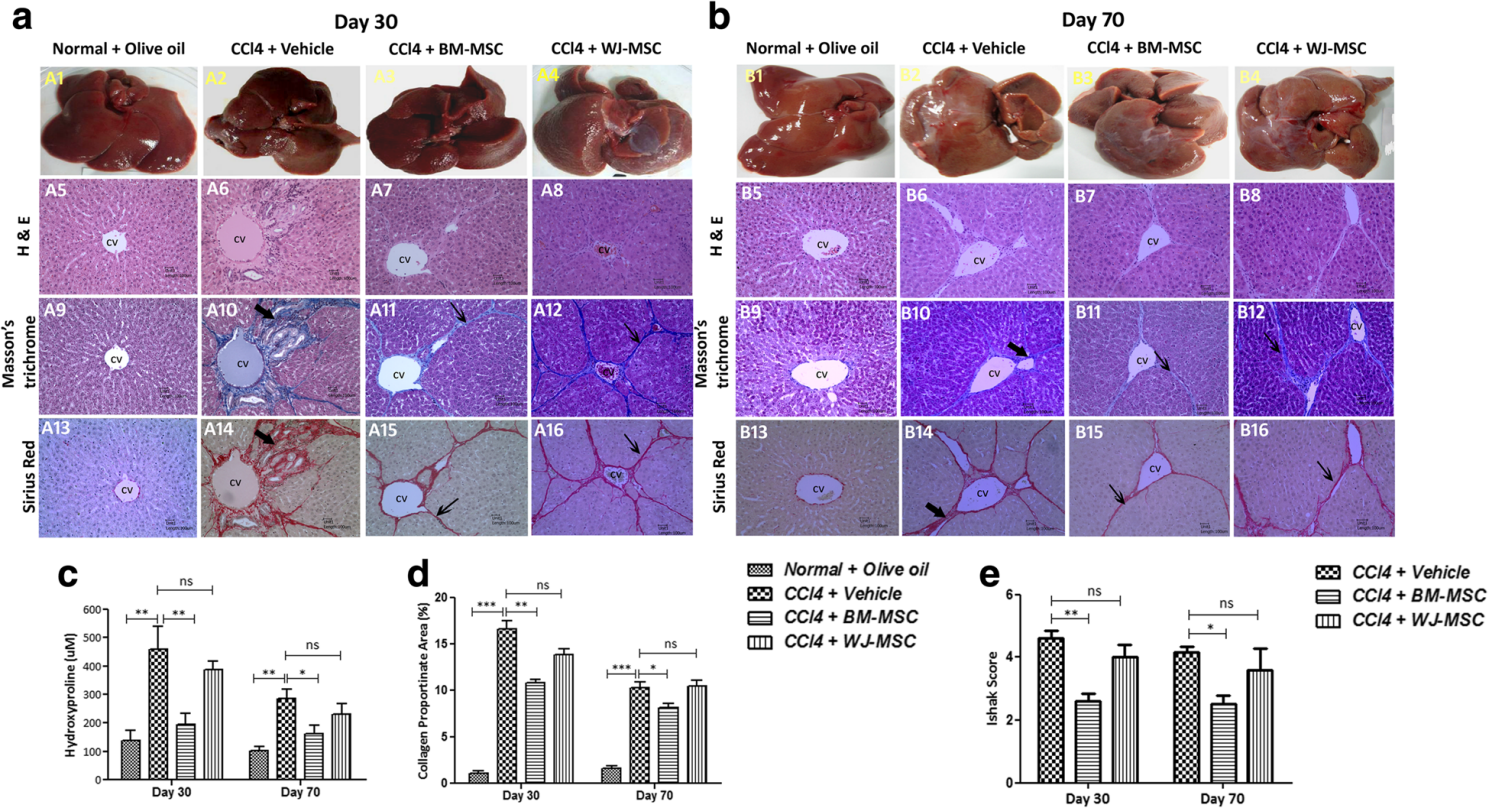
Histopathological analysis of liver tissue sections after transplantation of BM-MSCs and WJ-MSCs in CCl_4_-induced liver fibrosis/cirrhosis. **a**, **b** Representative images showing the gross morphology of liver (respective panel numbers 1–4,) and photomicrographs of liver tissue sections stained with hematoxylin and eosin (*H&E*; respective panel numbers 5–8), Masson’s trichrome (respective panel numbers 9–12), and Sirius red (respective panel numbers 13–16). **c** Quantitative analysis of hepatic hydroxyproline content on days 30 and 70. **d** Collagen proportionate area quantification by computer-assisted image analysis on days 30 and 70. **e** Histopathological analysis of liver sections using Ishak scoring criteria [26]. *Thick arrows* show thick strands bridging fibrosis; *thin arrows* show thin strands bridging fibrosis. *Scale bars* = 100 μm, magnification 10×. ***p < 0.001, **p < 0.01, *p < 0.05. *BM-MSCs﻿*; bone marrow-derived mesenchymal stromal cells, *ns*; not significant, *WJ-MSCs﻿*; Wharton’s jelly-derived mesenchymal stromal cells

As expected, the hydroxyproline levels in the liver samples of vehicle-treated animals were significantly higher than normal controls (Fig. 6c). Intravenous administration of BM-MSCs significantly reduced the hepatic hydroxyproline level both at day 30 (*p* < 0.01) as well as at day 70 (*p* < 0.05). Hydroxyproline levels were also reduced in WJ-MSC-treated animals; however, the magnitude of reduction was not significant as compared to vehicle-treated animals. These results further substantiated that BM-MSCs were more effective in reducing liver fibrosis than WJ-MSCs.

### Effect of BM-MSCs and WJ-MSCs on gross and microscopic liver histopathology

In comparison to normal control rats, the livers of vehicle-treated rats were enlarged, coarser, nodular on the surface, and liver lobes were fused with each other and to the peritoneal organs (Fig. 6a and b) at days 30 and 70. In contrast, rats treated with BM-MSCs showed less coarse surface at day 30 and became reddish, smoother, and lustrous at day 70 (Fig. 6b). Rats treated with WJ-MSCs showed marginal improvement. Histopathological analysis revealed that the Masson’s trichrome- and Picrosirius red-stained collagen fiber area was reduced in BM-MSCs- and WJ-MSCs-treated animals; however, the former population exhibited distinctly enhanced reduction in collagen fiber area compared to either the WJ-MSCs- or vehicle-treated sample. Furthermore, we quantified the Picrosirius red-stained area to analyze the collagen proportionate area. The percentage of collagen proportionate area was significantly reduced in BM-MSCs -treated animals as compared to vehicle-treated animals on day 30 (*p* < 0.01) and day 70 (*p* < 0.05). There was no significant reduction observed in the WJ-MSCs-administered samples (Fig. 6d). Furthermore, analysis of the Ishak fibrosis score showed a statistically significant reduction in mean score in BM-MSCs-treated animals as compared to vehicle-treated animals at both time points (Fig. 6e). Consistent with other data, WJ-MSCs-treated animals did not show such improvement.

### Immunohistochemical analysis of liver samples

**Fig. 7:**
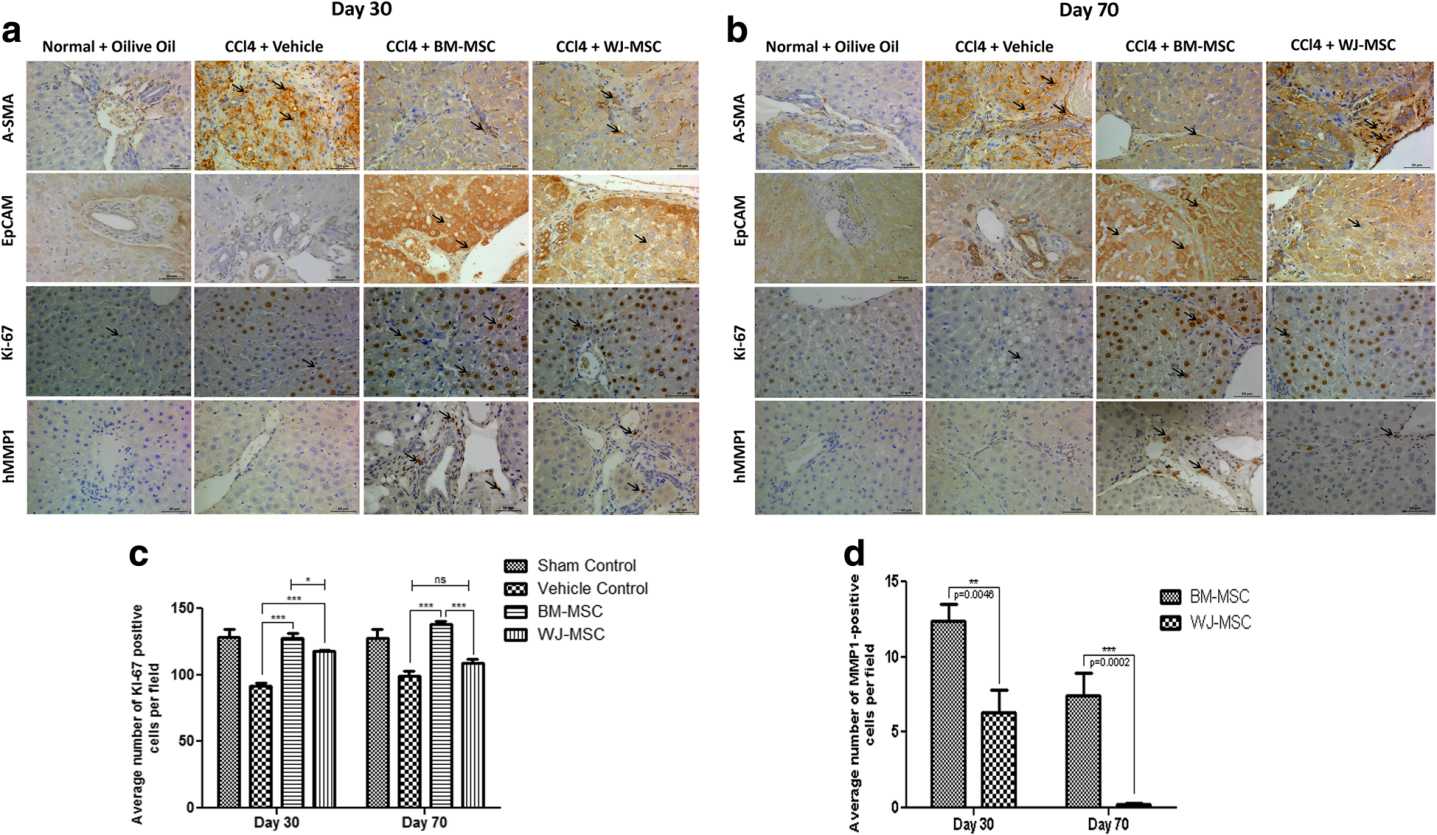
Immunohistochemical analysis of liver tissue sections after transplantation of BM-MSCs and WJ-MSCs in CCl_4_-induced liver fibrosis/cirrhosis. **a**, **b** Photomicrographs of liver tissue sections showing immunohistochemical staining for α-SMA indicating the activated myofibroblast, Ki-67 depicting cell proliferation, epithelial cell adhesion molecule (*EpCAM*) indicating the presence of hepatic stem/progenitor cells, and human MMP1 showing synthesis of metalloprotease-1. **﻿﻿c**, **d** Quantification of Ki-67 and MMP1 positive cells was performed at days 30 and 70 by counting the number of positive cells for five randomly selected fields﻿ in﻿ each group (n=3 rats for each time points). *P<0.05, ***p<0.001, ns; not significant.﻿﻿ *Scale bars* = 50 μm, magnification 40×. *Arrow heads* indicate immunohistochemically stained cells. *A-SMA* alpha-smooth muscle actin, *BM-MSCs﻿*; bone marrow-derived mesenchymal stromal cells, *hMMP1*; human matrix metalloprotease-1, *ns*; not significant, *WJ-MSCs*; Wharton’s jelly-derived mesenchymal stromal cells

To evaluate the effect of BM-MSCs and WJ-MSCs on activation of HSCs and myofibroblasts, α-SMA-positive cells were examined in the liver sections of vehicle- and MSCs-treated animals. Immunohistochemical staining of liver sections showed intensely stained α-SMA^+^ cells along the fibrous septa in the vehicle-treated animals (Fig. 7a and b) and the expression was markedly reduced in BM-MSC-treated animals at both days 30 and 70. Our data indirectly suggested that BM-MSCs reduced HSCs and fibroblast/myofibroblast activation in the liver fibrosis model. To determine whether transplanted MSCs promote hepatocyte proliferation and liver regeneration, the number of Ki-67^+^ cells were enumerated in five random fields (*n* = 3). The results showed that the number of Ki-67^+^ nuclei was highest in BM-MSCs-injected animals, followed by the WJ-MSCs- and vehicle-injected groups at days 30 and 70 (Fig. 7c). Similarly, more number of EpCAM^+^ cells were observed around the portal and ductal region in BM-MSCs-treated liver samples compared to WJ-MSCs and vehicle-treated groups, suggesting that BM-MSC administration may have induced proliferation of liver progenitor cells. Finally, we also examined the expression of the fibrolysis marker MMP1 and found that the number of human MMP1^+^ cells were higher in BM-MSCs-treated animals in comparison to those treated with WJ-MSCs. Thus, the results suggest that the BM-MSCs reduce fibrogenesis by secreting fibrolytic metalloproteases such as MMP1 and MMP2 (Fig. 7d).

## Discussion

Liver fibrosis is a complex and dynamic process that is orchestrated by various cell types and growth factors resulting in excessive accumulation of collagen and other extracellular matrix (ECM) proteins eventually leading to liver cirrhosis and hepatic failure. Although antifibrotic treatments are available and are commonly used, they are not very effective because they address a single specific mechanism of disease pathophysiology. Thus, the discovery and combination of new drugs that can target multiple cellular pathways of liver fibrogenesis are required for efficacious treatment.

Cell therapy is intended to treat through a multifactorial effect, and thus provides an ideal alternative to currently available treatments. MSCs are considered to be a potential candidate to ameliorate hepatic fibrosis because of their multiple therapeutic effects. Several in vitro studies have shown that MSCs derived from various sources can differentiate into hepatocyte-like cells when differentiation is induced by HGF and FGF4 [27]. Broadly, therapeutic activity can be attributed to secretion of many growth factors including VEGF and HGF and other cytokines, as well as various types of collagen-degrading MMPs. Immunosuppression and anti-inflammatory properties mediated via secretion of IDO, PGE2, IL10, etc. play an important role in reducing excessive inflammation and apoptosis [28].

In the current study, the comparative analysis of pooled BM-MSCs and WJ-MSCs was carried out in vitro. We have deciphered one of the mechanisms of action of both cell types in reversing the fibrosis using activated HSCs that are known to produce very high levels of collagen [29]. Our in vitro data suggest that secretome of BM-MSCs is superior in degrading collagen compared to that of WJ-MSCs. Furthermore, when we evaluated the expression of MMPs, BM-MSCs expressed a wide range of MMPs such as MMP1, -2, -13, and -16 that are known to catabolize the fibrotic collagens. In addition, the in vivo data demonstrated that intravenous infusion of BM-MSCs exhibited a greater potential to reduce liver collagen content and improve liver architecture as evidenced by liver hydroxylproline levels, collagen proportionate area, and Ishak score as compared to the WJ-MSCs population. The antifibrotic potential of BM-MSCs and WJ-MSCs has been demonstrated in various preclinical models of liver fibrosis [30], although no direct comparison between the two cell types has been performed in the same animal model. The comparative in vitro and in vivo efficacy results presented here suggest the BM-MSCs might be more effective due to a broader expression of various MMPs necessary for fibrous collagen degradation.

Growth factors such as VEGF and HGF secreted by MSCs are reported to reduce hepatocyte apoptosis and increase liver regeneration [31]. In the current study, BM-MSCs showed higher levels of VEGF secretion whereas WJ-MSCs secreted higher levels of HGF (Fig. 3b and c). Our data corroborates with earlier findings by Amable et al. [32] who demonstrated that BM-MSCs had higher potential to secrete VEGF compared to WJ-MSCs and adipose tissue-derived MSCs, and that WJ-MSCs secreted higher amounts of HGF compared to the other two cell types. Our study showed some difference in the MMP expression as compared to those performed by Amable et al. We believe these may be due to the use of different culture conditions such as the addition of bFGF and pooling of MSCs from three independent donors in our study. bFGF is known to regulate the MMP expression [33, 34]. Also, interestingly, it was shown that the migration of MSCs through bone marrow endothelium is regulated by MMP2 [35, 36] which showed higher expression in BM-MSCs. Our in vivo data show higher numbers of Ki-67^+^ and EpCAM^+^ cells in BM-MSCs-treated animals when compared to WJ-MSCs-treated animals (Fig. 7). It has been shown that both HGF and VEGF exhibit their antifibrotic effect by inhibiting HSCs activity [37] and by activating the CXCL9-MMP13 axis [38], respectively. Though WJ-MSCs secrete higher levels of HGF compared to BM-MSCs, WJ-MSCs showed reduced efficacy in ameliorating liver fibrosis. This suggests that higher amounts of HGF secretion alone is not sufficient to predict the potency of a particular MSC population to reduce CCl_4_-induced hepatic fibrosis. Immunofluorescence results for DiI/CD105^+^ cells showed that BM-MSCs persisted for a longer duration in the injured liver compared to WJ-MSCs. It is to be noted that Zhao et al. [39] demonstrated that intravenously transplanted BM-MSCs engrafted and rescued liver fibrosis more efficiently than intraperitoneal and intrahepatic routes of administration. Our study also confirmed that intravenous administration of BM-MSCs resulted in a marked reduction of liver fibrosis as compared to WJ-MSCs in our CCl_4_-induced liver fibrosis rat model.

## Conclusions

In conclusion, our data suggest that BM-MSCs are superior to WJ-MSCs in ameliorating CCl_4_-induced liver fibrosis in rats, which may be collectively due to several reasons: the differential capacity to home to the injured liver tissue; difference in paracrine activities; variation in MMP synthesis and secretion; and differences in inhibiting HSCs activation. Whether WJ-MSCs delivered through a different route can be as equally effective as BM-MSCs in treating fibrosis still remains an open question. Obviously, future studies would be required to compare antifibrotic effects of BM-MSCs with those derived from other tissues before selecting a cell-based therapeutic product to treat patients with liver fibrosis/cirrhosis.

## Abbreviations

bFGF: Basic fibroblast growth factor
BM: Bone marrow
BMMNCs: Bone marrow mononuclear cells
CCl_4_: Carbon tetrachloride
CM: Conditioned medium
EpCAM: Epithelial cell adhesion molecule
FBS: Fetal bovine serum
FGF: Fibroblast growth factor
HGF: Hepatocyte growth factor
HSC: Hepatic stellate cell
IDO: Indoleamine 2,3-dioxygenase
IL: Interleukin
MMP: Matrix metalloprotease
MSCs: Mesenchymal stromal cells
NGF: Nerve growth factor
P: Passage
PGE2: Prostaglandin E2
SMA: Smooth muscle actin
TGF: Transforming growth factor
VEGF: Vascular endothelial growth factor
WJ: Wharton’s jelly
